# Multivariate Optimization of Electrochemical Biosensors for the Determination of Compounds Related to Food Safety—A Review

**DOI:** 10.3390/bios13070694

**Published:** 2023-06-30

**Authors:** Héctor Fernández, María Alicia Zon, Sabrina Antonella Maccio, Rubén Darío Alaníz, Aylen Di Tocco, Roodney Alberto Carrillo Palomino, Jose Alberto Cabas Rodríguez, Adrian Marcelo Granero, Fernando J. Arévalo, Sebastian Noel Robledo, Gastón Darío Pierini

**Affiliations:** 1 Grupo de Electroanalítica (GEANA), Departamento de Química, Instituto para el Desarrollo Agroindustrial y de la Salud UNRC-CONICET (IDAS), Facultad de Ciencias Exactas, Físico-Químicas y Naturales, Universidad Nacional de Río Cuarto, Río Cuarto 5800, Argentina; hfernandez@exa.unrc.edu.ar (H.F.); azon@exa.unrc.edu.ar (M.A.Z.); smaccio@exa.unrc.edu.ar (S.A.M.); ralaniz@exa.unrc.edu.ar (R.D.A.); aylenditocco@gmail.com (A.D.T.); rcarrillo@exa.unrc.edu.ar (R.A.C.P.); jcabasrodriguez@exa.unrc.edu.ar (J.A.C.R.); agranero@exa.unrc.edu.ar (A.M.G.); farevalo@exa.unrc.edu.ar (F.J.A.); 2 Departamento de Tecnología Química (IDAS), Facultad de Ingeniería, Universidad Nacional de Río Cuarto, Río Cuarto 5800, Argentina

**Keywords:** electrochemical biosensors, chemometrics tools, design of experiments, multivariate optimization, food safety

## Abstract

We summarize the application of multivariate optimization for the construction of electrochemical biosensors. The introduction provides an overview of electrochemical biosensing, which is classified into catalytic-based and affinity-based biosensors, and discusses the most recent published works in each category. We then explore the relevance of electrochemical biosensors for food safety analysis, taking into account analytes of different natures. Then, we describe the chemometrics tools used in the construction of electrochemical sensors/biosensors and provide examples from the literature. Finally, we carefully discuss the construction of electrochemical biosensors based on design of experiments, including the advantages, disadvantages, and future perspectives of using multivariate optimization in this field. The discussion section offers a comprehensive analysis of these topics.

## 1. Introduction

The introduction section is divided into three parts. First, a brief introduction to electrochemical biosensors is provided. Second, we discuss the importance of electrochemical biosensors in ensuring the safety of food and food products. Finally, a comprehensive discussion on the application of multivariate optimization in the construction of electrochemical sensors/biosensors is presented.

### 1.1. Electrochemical Biosensors in Food and Food Product Safety

The development of electrochemical biosensors is primarily focused on the field of clinical diseases and, more recently, on chemistry, the environment, pharmaceutics, and food safety. Particularly, food safety has become one of the fundamental aspects of humanity due to the high contamination rate caused by human activities. As a result, there is a great need for the development of reliable and accurate methods for determining the quality of food worldwide [1]. In this context, electrochemical biosensors have a relevant place, due to their simplicity, sensitivity, and selectivity among others [2]. Several reviews on the development of biosensors for ensuring food safety control exist in the literature. These reviews discuss various approaches, including fast detection and affinity-based methods [3,4], detection of allergens and adulterants [5,6], aptasensors [7,8], nanomaterials employed in the construction of biosensors [9,10,11], metal–organic frameworks as biosensing platforms [12], and nanoenzyme-based biosensors [13], among many other very interesting reviews [14,15,16,17,18,19,20].

**Figure 1:**
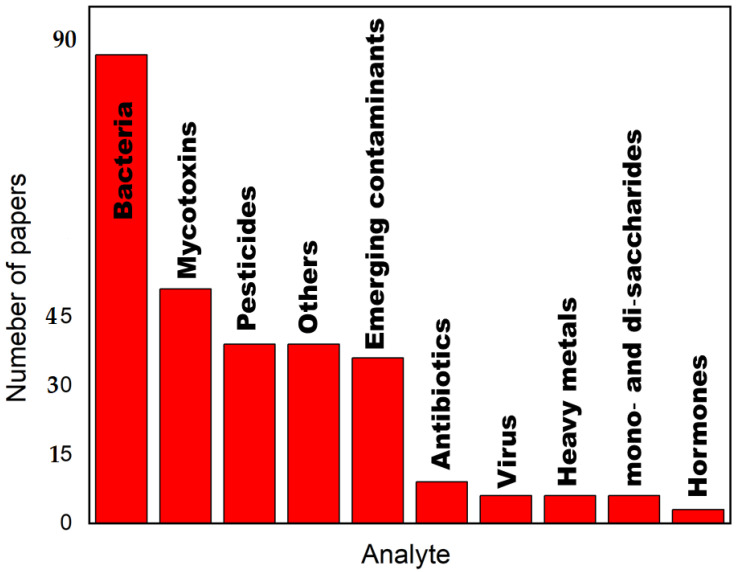
Schematic representation of the number of papers published for developed electrochemical biosensors according to the analyte related to food safety.

A Scopus search for the keywords “food safety” and “electrochemical biosensors” yielded a total of 279 articles. From this search, several analytes stand out as important considerations, such as mycotoxins (ochratoxin A, aflatoxin B_1_, etc. [21,22,23,24]), bacteria (*Escherichia coli*, Salmonella, Staphylococcus aureus, etc. [25,26,27]), and viruses (Vibrio parahaemolyticus, influenza virus, hepatitis A, etc. [28,29,30]), among others. Figure 1 shows the number of articles analyzed according to analyte.

As can be seen in Figure 1, bacteria (particularly *Escherichia coli*) are the primary group of analytes considered for ensuring food safety, accounting for over 30% of the papers found in the literature. Several electrode configurations were used for constructing electrochemical biosensors, including reduced graphene oxide, gold nanotube arrays, fullerene (C60), and many others [31,32]. A second group of analytes that should be seriously considered as indicators for food safety are mycotoxins, which account for 18% of the papers. Mycotoxins are toxic substances that often contaminate food and food products, and their presence has severe implications for human health [33,34]. A third group of analytes considered for ensuring food safety are pesticides, emerging contaminants (synthetic or naturally occurring chemicals or any microorganisms that are not commonly monitored in the environment but have the potential to enter the environment and cause known or suspected adverse ecological and/or human health effects), and other compounds (chemicals which do not fit into the other categories mentioned, but are presented in the literature as indicators of food quality) such as L-glutamate, 6-benzyladenine, and carbaryl, among others [35,36,37]), with a concentration range that varied between 12 and 13%. These groups of compounds, particularly pesticides, have gained a lot of importance in recent years, and many studies have been developed regarding these analytes [16,38,39]. The last group of analytes, which includes antibiotics, viruses, heavy metals, mono- and di-saccharides, and hormones, represents a smaller portion (between 1 and 3%) of the electrochemical biosensors developed to food safety, accounting for a total of 10% of the published papers on this topic. Therefore, an ultrasensitive immuno-sensor has been developed for the detection of Penicillin G in food samples. The immuno-sensor was based on reduced graphene oxide (rGO) and was used to detect an electrode conjugated with anti-Penicillin antibody [40]. To detect hepatitis A virus, an electrochemical biosensor based on disposable gold electrode functionalized with the specific capture probe and tested on complementary ssDNA has been developed [30]. A new electrochemical biosensor has been developed to detect Pb^2+^ based on GR5 DNAzyme/Ti_3_C_2_Tx Mxenes modified with a glassy carbon electrode (GR5 DNAzyme/Ti_3_C_2_Tx Mxenes-GCE) [41]. Glucose detection is vital in the field of food safety. Therefore, a hydrogel-based electrochemical biosensor was proposed to detect glucose via electrochemical impedance spectroscopic (EIS) measurements [42]. An electrochemical biosensor based on graphitic carbon nitride (g-C_3_N_4_) and a conductive polymer 3-aminopropyltriethoxysilane (APTES) was developed to carry out the selective and sensitive determination of hormone 17β-estradiol [43].

### 1.2. Electrochemical Biosensors

**Figure 2:**
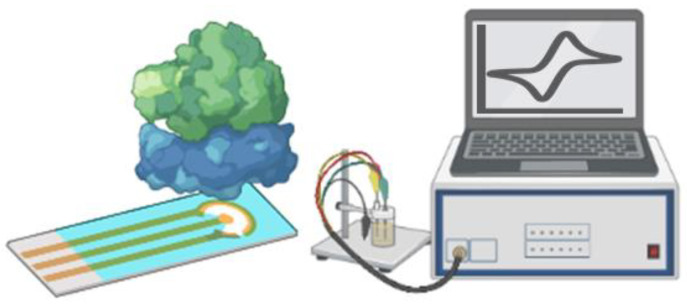
A typical design of an enzyme modified electrochemical biosensor.

Electrochemical biosensors are sensing devices that transduce biochemical events into electrical signals. According to the definition of the International Union of Pure and Applied Chemistry (IUPAC), an electrochemical biosensor is a self-contained integrated device capable of providing specific quantitative or semi-quantitative analytical information using a biochemical receptor in direct spatial contact with an electrochemical transduction element [44]. For example, a biochemical event could be an enzyme–substrate reaction or antigen–antibody interaction, among others, and the electrical signal could be measured as a change in current, voltage, impedance, etc. [45]. Electrochemical biosensors are recognized as reliable and typically portable tools for the rapid and cost-effective determination of a wide range of analytes, including metal ions, organic compounds, pollutants, proteins, antigens, deoxyribonucleic acids (DNAs), viruses, bacteria, and others [46]. Particularly, biosensors based on enzymes are well known [47]. Briefly, we can find three kinds of enzyme-based electrochemical biosensors in the literature. First-generation biosensors are based on oxygen. The second-generation biosensors are based on the use of redox mediators. The third-generation biosensors are those in which the enzyme is directly coupled on the electrode surface [48]. Very recently, Sumitha and Xavier [49] proposed definitions for fourth- and fifth-generation electrochemical biosensors. However, these are very recent advances, which are beyond the scope of this review. Figure 2 displays a typical design of an enzyme-modified electrochemical biosensor.

The electroactivity of a substrate or product of an enzymatic reaction generates the basis of first-generation enzymatic biosensors [50]. Second-generation biosensors utilize redox mediators, which are small electroactive molecules that transport electrons between the enzyme active sites and an electrode, through mediated electron transfer (MET) mechanism. [51,52]. Direct electrical communication between the electrode and the redox center of the enzyme characterizes third-generation biosensors. These biosensors offer several advantages, including a superior selectivity, sensitivity, reagentless detection, and label-free fabrication [53].

The central part of an electrochemical biosensor is the electrode, which acts as a solid support for the immobilization of various biomolecules such as enzymes, antibodies, and nucleic acids, as well as for electron transfer. The construction of an electrochemical biosensor involves several steps, typically including electrode preparation, electrode modification (often with nanostructures), and biological element immobilization [52,54,55,56].

**Scheme 1:**
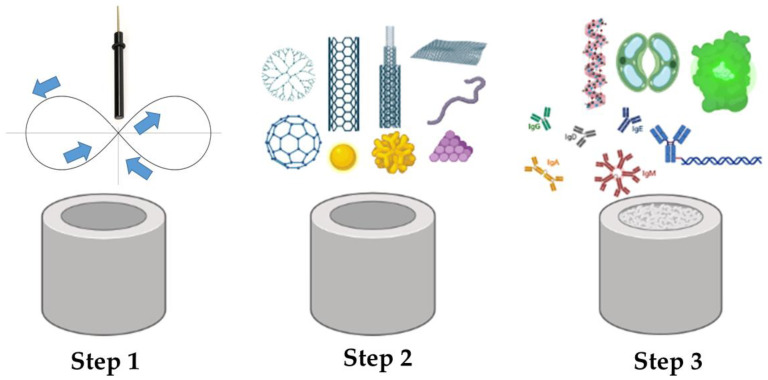
Sequential steps for the construction of an electrochemical biosensor using a glassy carbon electrode.

Various types of working electrodes have been used to develop electrochemical biosensors, including glassy carbon, gold, platinum, and screen-printed electrodes, among others [13,14,15,16,17,18]. The choice of working electrode material typically involves different electrode preparations. For example, a glassy carbon electrode is typically polished with different sizes of alumina (step 1 in Scheme 1) [56]. On the other hand, a screen-printed electrode surface can be electrochemically pre-treated with KOH aqueous solution by applying a positive potential step during a certain time, following a procedure described by Anjo et al. [57].

Once the electrode surface has been conditioned, the next step is to modify the surface with nanostructures (step 2 in Scheme 1). Various materials, such as multi-walled carbon nanotubes, copper oxide nanoparticles, graphene oxide, gold nanoparticles, and others, have been used to modify electrodes and improve their performance. [52,58,59,60,61,62].

The attractiveness of such nanomaterials relies not only on their ability to act as effective immobilization matrices, but they also have the potential to enhance the long-term stability of electrochemical biosensor devices. These matrices possess unique features such as large surface areas, controlled morphology and structure, and electrocatalytic properties that can be effectively combined with biomolecules to improve the sensitivity and selectivity of electrochemical biosensors [63].

The third step, as it is described in Scheme 1, involves the immobilizing of the biorecognition element. Enzymes, antibodies, cells or tissues, which possess high biological activity, can be immobilized at the surface of the transducer using various procedures. These include entrapment behind a membrane, entrapment within a polymeric matrix, entrapment within self-assembled monolayers (SAMs), covalent bonding on surfaces or membranes activated by bifunctional groups or spacers, and bulk modification of the entire electrode material [33,34,52,54,55,56,60,64,65,66,67,68,69,70]. Electrochemical biosensors commonly used are of three types of recognition elements: (1) enzymes, which are the most common and well-developed recognition system, and can be either mono- or multi-enzyme-based; (2) whole cells (microorganisms such as bacteria, fungi, eukaryotic cells, or yeast) or cell organelles or particles (such as mitochondria and cell walls); and (3) tissues (such as plant or animal tissue slices) [71].

As can be seen, the construction of an electrochemical biosensor involves several steps and requires the analysis of many factors [72].

### 1.3. Multivariate Optimization

Therefore, the most common method for optimizing analytical methods is the “one factor at a time” (OFAT) approach. While this method requires significant experimental work and only provides local optima, it does not take into consideration possible interactions among the factors being tested. As a result, it often leads to suboptimal results compared to multivariate optimization [73].

Chemometrics is a discipline that utilizes statistical and mathematical fundamentals to analyze data from different chemical or physical interactions and to find correlations between variables [74]. One of the most important chemometric tools is called “the experimental design”. A multivariate experimental design is a type of experimental research that includes the study of more than one dependent variable at the same time [75].

Although experimental design has been a well-known tool for a long time, it has been underutilized in the development of electrochemical biosensors [76]. However, there are some examples where experimental design is combined with the development of electrochemical sensors. Robledo et al. [77] used a Box–Behnken design (BBD) to optimize experimental variables to generate glassy carbon electrodes (GCE) modified with electrochemically partially reduced graphene oxide (GCE/ePRGO) to study the caffeic acid (CA) electrochemical oxidation. The independent variables selected for the optimization were: the scan rate, the number of cycles, and the volume of the drop of the dispersion of GO and the dilution of the dispersion of GO in water. After the numerical optimization, 0.052 V s^−1^; 30; 10 × 10^−3^ cm^3^; and 0.66 (GO volume (cm^3^): H_2_O volume (cm^3^)) were the optimal values, respectively. On the other hand, a BBD combined with desirability function was used for the optimization of square wave parameters. The studied factors were as follows: step potential (ΔE_s_), frequency (f), and amplitude of the square wave (ΔE_sw_). In order to optimize the best square wave parameters for the simultaneous determination of zinc, cadmium, lead, and copper, the desirability function was used. Under these conditions, the optimal values obtained were: ΔE_s_ = 3 mV, ΔE_sw_ = 70 mV, and f = 10 Hz [78]. Krepper et al. [79] carried out the determination of tetracyclines in honey samples by using an “in situ” antimony film electrode. The optimization of adsorptive cathodic stripping voltammetry and SWV parameters (SWCSV) has been carried out by using the Draper Lin small composite design. For this, the deposition time (t_d_), ΔE_SW_, f and ΔE_s_ were studied. The optimum SWCSV parameters were ΔE_s_ = 4 mV, ΔE_sw_ = 160 mV, f = 130 Hz, and t = 7 s. Simultaneous determination of hypoxanthine, xanthine, and uric acid in fish samples were carried out by using SWV and an edge plane pyrolytic graphite electrode (EPPGE). A composite central design was used to optimize the pretreatment of the working electrode. In addition, the same design to optimize the accumulation step (accumulation time and potential) was used [80]. The accumulation step for taxifolin determination at graphite screen-printed electrodes was optimized by using a composite central design [81].

As can be seen from the few examples shown, experimental design is widely used for the optimization of electrochemical sensors in many aspects, such as the optimization of electrochemical technique parameters [82,83,84,85], the composition of the surface modification [86,87,88,89], and accumulation parameters [90,91,92,93], among several other examples. The most common designs used to obtain the response surface in the mentioned examples are Box–Behnken and central composite.

The Box–Behnken experimental design (BBD) explores and models system or process responses using three or more continuous independent variables. Thus, its goal is to find the optimal combination of variable levels that maximize or minimize a desired response. This design strategically selects experimental points within defined ranges for each variable and uses second-order response surfaces to model the relationships. The BBD employs a matrix of points arranged in a spherical or ellipsoidal pattern, carefully chosen to minimize correlation between the independent variables and estimate second-order model coefficients. Advantages of the BBD include efficiency, good coverage of the design space, second-order response surfaces, and easy interpretation through visualizations. Disadvantages of the Box–Behnken design include its limitation to three variables and the dependence on center points for accuracy.

A Central Composite Design (CCD) is a statistical technique used to study the relationship between independent variables and the response in an experiment. This design combines a fractional factorial design with additional center points to explore and model the responses while determining the main effects and interactions of the variables. In a CCD, low, high, and central values are chosen for each independent variable. The central values assess linearity and possible response curvature, while the high and low values assess the effects of variable extremes. Axial points, located further away from the center values, are included in the design to evaluate non-linearity effects and detect optimal or high-performance points. Once experiments based on the CCD are performed, a mathematical model is fitted to describe the relationship between the independent variables and the response. This model enables predictions and optimization of the desired response. Advantages of the CCD include flexibility in the number of variables, detection of curvature effects, and increased modelling accuracy. Disadvantages of the CCD include the requirement for a larger number of experiments and the need for prior knowledge of variable limits for proper selection of axial points.

Overall, the choice between BBD and CCD depends on the number of variables, available resources, and the complexity of the system or process under study [94].

However, despite the existence of several examples of multivariate optimization for the development of electrochemical sensors, there are not many examples found in the literature for electrochemical biosensors. The next section (Section 2) describes in detail the examples found in the literature about the multivariate optimization for the construction of electrochemical biosensors for food control.

## 2. Multivariate Optimization in Electrochemical Biosensors

### 2.1. Summary of Optimized Biosensors by Response Surface Methodology

In this section, we will present a review of the most prominent scientific papers in the development of electrochemical biosensors applying experimental design with response surface methodology for the optimization of electrochemical response to determine related compounds to food safety.

One of the main highlights of the articles is the use of multivariate optimization techniques to improve the performance of biosensors. This approach considers the interactions among multiple variables to identify the optimal conditions for biosensor construction, leading to more efficient and sensitive biosensors. Additionally, the use of electrochemical biosensors as an analytical tool for food safety is a promising and growing field due to its sensitivity, selectivity, and rapid detection capabilities.

In the publication titled “A novel non-enzymatic glucose sensor based on the modification of carbon paste electrode with CuO nanoflower: Designing the experiments by response surface methodology (RSM),” Z. Amani-Beni and A. Nezamzadeh-Ejhieh [95] reported the development of a non-enzymatic electrochemical biosensor for glucose detection using a CuO nanoflower-modified carbon paste electrode (CuO-CPE) and RSM. The authors employed RSM to optimize the experimental factors, including CuO percentage, pH, amplitude, step potential, and frequency. The optimal conditions were achieved with 20% CuO, pH 3.6, amplitude 0.106 V, step potential 0.0074 V, and frequency 17.75 Hz. The biosensor showed a linear response range from 0.06 to 10 mmol L^−1^, with detection and quantification limits of 7.49 × 10^−10^ and 2.49 × 10^−9^ mol L^−1^, respectively. Moreover, the CuO-CPE biosensor showed good performance in the determination of glucose in human blood serum samples.

M. Darvishi et al. [96] reported a study titled “Surface blocking of azolla modified copper electrode for trace determination of phthalic acid esters as the molecular barricades by differential pulse voltammetry (DPV): response surface modelling optimized biosensor”. In this study, they investigated the development of a voltammetric biosensor for detecting phthalic acid esters (PAEs) in water. The biosensor is based on modifying a copper electrode with azolla paste made using azolla powder and electroencephalography gel. The study used a central composite design (CCD) to optimize experimental parameters, resulting in predicted optimal conditions for concentration of Fe^2+^, supporting electrolyte, pH, and modifier/gel mass ratio. Linear relationships were found between the DPV responses and PAEs concentrations, with a limit of detection (LOD) and a limit of quantification (LOQ) in the ranges of 0.2–0.4 μg L^−1^ and 0.5–1.0 μg L^−1^, respectively. Thus, the study suggests that this method is efficient, accurate, and quick for the determination of PAEs in real water samples.

Díaz Nieto et al. [65] reported a paper titled “Development of a third-generation biosensor to determine sterigmatocystin mycotoxin: An early warning system to detect aflatoxin B1”. In this study, the authors developed a third-generation enzymatic biosensor to detect sterigmatocystin (STEH) by modifying a glassy carbon electrode with soybean peroxidase enzyme (SPE) and chemically reduced graphene oxide. The biosensor was optimized using an RSM. The biosensor showed a linear response in the concentration range from 6.9 × 10^−9^ to 5.0 × 10^−7^ mol L^−1^, with a limit of detection of 2.3 × 10^−9^ mol L^−1^. The biosensor was used to detect STEH in corn samples spiked with STEH, with an average recovery of 96.5%, and also in corn samples inoculated with the Aspergillus flavus fungus. The decrease in STEH over time was related to the production of aflatoxin B1 (AFB1). Results obtained with the biosensor were in good agreement with those obtained by HPLC. The apparent Michaellis–Menten constant (KM-M_app_) was (1.5 ± 0.2) × 10^−6^ and (1.2 ± 0.2) × 10^−6^ mol L^−1^ using both the Lineweaver–Burk and Eadi–Hofstee methods, respectively.

A. Dwevedi et al. [97] reported a paper titled “Lactose nano-probe optimized using response surface methodology.” This study was focused on the development of a lactose nano-probe by immobilizing Pisum sativum enzyme lactase (PsBGAL) on gold nanoparticles (AuNps) using a spacer arm (cysteamine–glutaraldehyde). RSM was used to optimize the immobilization, resulting in an efficiency of 140.81%. The AuNps-PsBGAL was characterized and was found to exhibit a broad temperature and pH optima, as well as a significant increase in catalytic efficiency when compared to soluble PsBGAL. The immobilized enzyme was stable under dried conditions for 6 months and was reusable for over five batchwise uses without loss of activity. The Hill’s coefficient was found to be 1.71, corresponding to a concentration range of lactose from 0.1% to 2.0%. This nano-probe can be useful for people with severe lactose intolerance for use in quality checks of lactose-hydrolyzed milk and detection of hidden lactose in various food products.

M. D. Gouda et al. [98] developed an immobilized biosensor for the electrochemical detection of sucrose in food and fermentation samples. The biosensor used a multienzyme system of invertase, mutarotase, and glucose oxidase (GOD), immobilized using glutaraldehyde. RSM was used to optimize the operating parameters, with invertase at 10 IU, mutarotase at 40 IU, and GOD at 9 IU found to be the optimal conditions. The biosensor had a response time of 2.35 min and showed good agreement with the predicted response time of 2.26 min. The range of sucrose analyzed was 1-10%, and the optimal conditions achieved using the RSM method improved the response time of the biosensor.

L. Mirmoghtadaie et al. [99] reported a study, which describes an electrochemical DNA biosensor for detecting folic acid using a pencil graphite electrode modified with salmon sperm ds-DNA. RSM was used to optimize the immobilization of ds-DNA on the electrode, determining the best conditions for pH, DNA concentration, deposition time and deposition potential. The binding of folic acid to DNA was detected by measuring the electrochemical signal of adenine. The biosensor showed an LOD of 1.06 × 10^−8^ μmol L^−1^ and was successfully used to detect folic acid in different real samples. The biosensor showed low relative standard deviations for ten replicate differential pulse voltammetric measurements of 2.0 and 5.0 μmol L^−1^ folic acid, with values of 4.6% and 4.3%, respectively.

Sarika et al. [100] reported the development of an electrochemical biosensor for detecting disubstituted methyl and methoxy phenols using immobilized laccase enzyme from Trametes versicolor. In this study, three immobilization methods were compared, and the crossed-linking method with bovine serum albumin (BSA) on nylon membrane was found to be the best. The concentrations of laccase, BSA, and glutaraldehyde were optimized using the Box–Behnken design to increase the sensitivity of the biosensor. The biosensor operated based on an amperometric principle, and the immobilized enzyme laccase was sensed electrochemically. The optimization of parameters resulted in improved sensitivity, LOD, response time, and operating stability.

N. Talib et al. [101] reported the development of an immunosensor for the detection of Clenbuterol (CLB), an illegal antibiotic for livestock. The immunosensor was modified with poly(3,4-ethylenedioxythiophene) (PEDOT), multi-walled carbon nanotubes (MWCNT), and anti-CLB antibody (Ab) using a screen-printed carbon electrode (SPCE) as the sensor platform. A competitive-type immunoassay was performed to specifically bind CLB with Ab. The electrochemical immunoassay conditions such as pH, incubation temperature, antigen (Ag) incubation time, and % blocking agent were optimized using the response surface methodology/central composite design (RSM/CCD) to obtain a high current signal. The developed immunosensor was highly reproducible and sensitive, with good storage stability. The study demonstrated that the immunosensor showed comparable results with those of liquid chromatography-mass spectrometry in real meat samples, making it useful for CLB screening and monitoring.

De Benedetto et al. [102] reported the optimization of an electrochemical biosensor for the detection of metal ions using RSM and CCD. The biosensor was optimized for enzyme concentration, flow rate, and number of cycles. The optimized biosensor showed a sensitivity of 50 U mL^−1^, 30 scan cycles, and 0.3 mL min−1 flow rate. The biosensor was tested for its response to Bi^3+^, Al^3+^, Ni^2+^, and Ag^+^ ions and had a wide working range and high reproducibility (RSD = 0.72%). This study reports for the first time the Bi^3+^ and Al^3+^ inhibition on the Pt/PPD/GOx biosensor response.

Urkut et al. [103] discussed the optimization of foodborne pathogen detection using label-free electrochemical nucleic acid biosensors through the application of RSM. The study optimized the concentration of probes, hybridization time, and LOD of biosensors for the detection of Listeria monocytogenes in real samples. The results showed that the biosensor has a sensitivity of 2.2 × 10^−6^ mol/L, a linear concentration range from 1 × 10^−5^ to 1 × 10^−11^ mol/L, and an LOD of 7 × 10^−12^ mol/L. This paper also provides an overview of the progress and application of impedimetric biosensors for the detection of foodborne pathogenic bacteria, with a focus on new trends, such as the use of specific bio-recognition elements like bacteriophages and lectin, as well as nanomaterials and microfluidics techniques. The importance of developing a rapid, sensitive, and specific method for detecting foodborne pathogenic bacteria for ensuring food safety and security is emphasized.

B. Dalkıran [104] developed a new biosensor for detecting heavy metals based on horseradish peroxidase enzyme inhibition. The biosensor used a glassy carbon electrode (GCE) modified with indium tin oxide (ITO) nanoparticles, hexaammineruthenium (III) chloride (RUT), and chitosan (CH) as the working electrode. The electrode composition was optimized using a CCD. The biosensor showed a good sensitivity, with an LOD of 8 nM, 3 nM, and 1 nM for Pb^2+^, Ni^2+^, and Cd^2+^, respectively. The biosensor response was found to be within the range of 0.009–0.301 µM for Pb^2+^, 0.011–0.368 µM for Ni^2+^, and 0.008–0.372 µM for Cd^2+^. The type of HRP inhibition by heavy metals was investigated using the Dixon and Cornish–Bowden plots. The proposed biosensor was capable of detecting Pb^2+^, Ni^2+^, and Cd^2+^ in tap water with satisfactory results that were in a good agreement with atomic absorption spectrometry.

Nandakumar et al. [105] developed a low-cost, small-sized electrochemical biosensor for detecting bacterial contamination using printed circuit boards. The biosensor was constructed using a combination of photo-lithography and electro-deposition and consisted of thin-film gold electrodes coated with bio-receptors to detect changes in electrical impedance caused by pathogen binding on the sensor surface. The sensor geometry was optimized using experimental design techniques, and the device could be operated using a small excitation potential of magnitude 5 mV. The biosensor was tested on the foodborne pathogen Salmonella typhimurium and successfully detected bacterial concentrations as low as 500 CFU/mL within 6 min.

Table 1 summarizes the previous papers in an easier and more visual way to compare different optimizations.

### 2.2. Advantages to Use RSM in Biosensors Construction

As seen in a previous section, few works have reported on the use of response surface methodology in constructing electrochemical biosensors. Optimization can be carried out at different steps of biosensor construction (see Scheme 1). Some optimizations were performed to improve the immobilization efficiency, minimize the biosensor response time, improve the immunoassay conditions, select the best electrochemical technique parameters, etc. The sensitivity of the electrochemical biosensor improves with each step that is optimized. However, only Diaz Nieto et al. (to the best of our knowledge) [65] used a general optimization of the construction of the biosensor (taking into account the different steps in its construction), selecting the most influential parameters by means of an Ishikawa diagram and then selecting their optimal values by RSM. Despite this, the aforementioned work did not include the variables corresponding to conditioning the electrode surface. Therefore, the authors believe in the importance of the development of an electrochemical biosensor where all stages of its construction are optimized by means of RSM, to achieve better figures of merit in the developed biosensor.

Biosensors optimized by RSM have several advantages over those built by OFAT, such as: faster response time, lower reagent consumption, better sensor design, higher sensitivity, lower detection limits, higher immobilization percentage of biological recognition elements, etc. All of these advantages were achieved with a relatively small number of experiments (typically around 30, taking into account the number of factors being studied). On the contrary, the biosensors developed by the OFAT method, although they study the factors involved in the construction of the biosensor, do not carry out a detailed analysis of the interactions between the factors. As a result, they achieve a local optimization of the factor rather than a global one. Typically, these studies involve multiple experiments (even more than when RSM is used), resulting in higher reagent consumption and longer biosensor development time.

## 3. Discussion

This comparative review summarizes different designs and performances of several electroanalytical biosensors. The reviewed biosensors demonstrate high sensitivity and specificity in detecting target analytes, showing potential for use in food safety. All the biosensors perform well at detecting target analytes in real samples, including water, corn, and other samples, with good recovery percentages and low RSD, indicating efficient, accurate, and quick methods. The studies aim to detect different analytes, including clenbuterol, metal ions, and pathogenic bacteria, using various bio-recognition elements such as enzymes, antibodies, and bio-receptors. Additionally, biosensors were made from various electrode materials, including screen-printed carbon electrodes, glassy carbon electrodes, and thin-film gold electrodes. The discussed biosensors demonstrate the potential use of response surface methodology and central composite design (except in one case, which used the Box–Behnken design) for optimizing biosensor parameters. Therefore, the studies suggest that the optimization of parameters using response surface methodology and the design of experiment method can lead to the development of efficient electroanalytical biosensors for to be used in the detection of various analytes.

It is important to emphasize that no biosensor has been developed (to the best of our knowledge) in which all steps of its construction have been optimized. This does not necessarily imply a higher consumption of biological recognition elements, since, for example, the electrode conditioning step (Step 1) can be optimized to generate functional groups on the electrode’s surface that allow or enhance the anchoring of nanostructures. Steps 2 and 3 (Scheme 1) are the ones that are commonly optimized using the RSM. However, the synthesis of nanostructures (e.g., gold nanoparticles, functionalization of carbon nanotubes, etc.) used in the construction of an electrochemical biosensor has not yet been optimized. This will reduce the consumption of reagents in the preparation steps and respect the principles of green chemistry.

It should be noted that there are very few papers where multivariate optimization was used in the development of electrochemical biosensors, as shown in the previous section. This shows that, although they are well-studied fields (chemometrics and sensor development), more efforts should be made to combine both strategies, which will result in better analytical developments.

The development of electrochemical biosensors for the determination of compounds related to food safety has advanced significantly in recent years, driven by the development of nanotechnology, nanomaterials, and new detection and data analysis methods. This review discussed and summarized recent progress in the biosensor design by using RSM. To achieve continuous improvement in sensitivity, selectivity, portability, and detection limits, the authors encourage the use of RSM (in all steps) to construct electrochemical biosensors to be used in food quality control.

## 4. Conclusions

Today, the most commonly used approach for optimizing analytical methods is the one-factor-at-a-time (OFAT) method, which only considers one variable while the rest remain constant. However, this approach can result in suboptimal outcomes when compared to multivariate optimization.

With the emergence of chemometrics, which applies statistical and mathematical principles to analyze data of different origin (chemical or physical), experimental design has become increasingly important.

Thus, this methodology, which involves the study of more than one dependent variable at a time, has been underutilized in the development of electrochemical biosensors. Nevertheless, there are some examples where it has been combined with the development of these sensors. In this review, we compare and evaluate the performance of electroanalytical biosensors that were optimized using experimental design, specifically through response surface methodology. By studying multiple parameters simultaneously, these biosensors can detect various analytes more efficiently. Our findings show that, by applying the optimizing of the characteristic parameters of these biosensors, they can detect target analytes in various real samples with high sensitivity and specificity, indicating their potential use in food safety.

## Figures and Tables

**Table 1:** Electrochemical biosensors optimized through response surface methodology for detection of compounds related to food safety.

Group of Analytes	Analytes	Electrochemical Technique	Experimental Design	Response	Factors	Number of Experiments	LOD/M	Sample	Advantages over non Optimized Biosensors	Reference
Mono- and di-saccharides	Glucose	Square wave voltammetry	Central composite design	Square wave voltammetric response	- CuO percentage - pH - Amplitude - Step potential - Frequency.	53	7.49 × 10^−10^	Human blood sample	- Very low LOD - Square wave parameters optimized - Electrode surface optimized - Selectivity	[95]
	Lactose	n.r.	Box–Behnken design	% immobilization efficiency	- Amount of AuNps - Cysteamine - Glutaraldehyde - Amount of enzyme	29	n.r.	n.r.	- High immobilization efficiency - Reduced enzyme consumption	[98]
	Sucrose	Amperometry	Box–Behnken design	Biosensor response time	- Concentration of invertase - Concentration of mutarotase - Concentration of glucose oxidase	15	n.r.	n.r.	- Fast biosensor response - Lowest concentration of all the enzymes	[97]
Emerging contaminants	Dibutyl phthalate (DBP), dimethyl phthalate (DMP), di(2-ethylhexyl)phthalate (DEHP) and dicyclohexyl phthalate (DCHP)	Differential pulse voltammetry	Central composite design	Decrease in the anodic peak current	- Fe^2+^ concentration - KCl - pH - Azolla modifier to gel mass ratio	30	- DBP: 7.18 × 10^−10^ - DMP: 1.03 × 10^−9^ - DEHP: 1.02 × 10^−9^ - DCHP: 6.05 × 10^−10^	Water	- Optimization of the electrode’s surface - Very low LOD	[96]
	2,6-dimethoxy phenol	Amperometry	Box–Behnken design	Maximum response in voltage of the biosensor	- Concentration of laccase - Concentration of BSA - Concentration of glutaraldehyde	15	0.3 × 10^−5^	Simulated industry effluents	- Optimization of the operational conditions of the biosensor	[100]
	Clenbuterol	Amperometry	Central composite design	Maximize the immunosensor current response	- pH - Incubation temperature - Antigen incubation time - % blocking	26	1.68 × 10^−8^	Fresh beef	- Optimization of the electrochemical immunoassay conditions - Optimization of the immunoassay can enhance the sensitivity of the sensor.	[101]
Heavy metals	Al^3+^ Bi^3+^	Amperometry	Central composite design	Maximize the sensitivity	- Enzyme concentration - Flow rates - Number of cycles were	20	- Al^3+^: 1.6 × 10^−5^ - Bi^3+^: 3.9 × 10^−6^	n.r.	- The performance of an amperometric biosensor was successfully maximized using an experimental design in terms of sensitivity	[102]
	Pb^2+^ Ni^2+^ Cd^2+^	Amperometry	Central composite design	Maximize the amperometric current	- Amount of ITO nanoparticles - Amount of RUT	13	Pb^2+^: 8.0 × 10^−9^ Ni^2+^: 3.0 × 10^−9^ Cd^2+^: 1.0 × 10^−9^	Tap water drinking	- Optimization of the electrode composition - Improved sensitivity	[104]
Bacteria	*Listeria monocytogenes*	Differential pulse voltammetry	Central composite design	Maximize the electrochemical signals obtained from H/NC (hybrid/non complementary)	- Target concentration - Hybridization time - Salt concentration	20	2.67 × 10^−10^	Several real food samples such as ice-cream, chicken, mayonnaise.	- Best biosensor sensitivity and specificity - Optimization of the hybridization parameters	[103]
	*Salmonella typhimurium*	Changes in electrical impedance	2^2^ factorial design	Resistances to electron transfer	- Total sensor length - Ratio of counter to reference electrode surface areas	5	500 CFU/mL	n.r.	- Optimization of the sensor geometry	[105]
Mycotoxins	Sterigmatocystin	Amperometry	Central composite design	Maximize the sensitivity	- Volume of both SPE and CRGO to be mixed - The volume of composite deposited at the electrode surface - Drying temperature of electrode - pH - Potential at which the amperometric measurements are performed	n.r.	2.3 × 10^−9^	Corn samples	- An Ishikawa diagram (or cause–effect diagram) was used to select the relevant factors to be studied - Biosensor development has higher sensitivity	[65]
Others	Folic acid	Differential pulse voltammetry	Central composite design	Variation of the electrochemical signal	- pH - DNA concentration - Time of DNA deposition - Potential of deposition	31	1.06 × 10^−8^	- Wheat flour - Spinach	- Immobilization of the ds-DNA electrode’s surface was optimized using response surface methodology. - A lower consumption of DNA was achieved to obtain a high sensitivity	[99]

n.r.: not reported.

## Data Availability

Not applicable.
